# Nanoparticle surface coatings produce distinct antibacterial effects that are consistent across diverse bacterial species

**DOI:** 10.3389/ftox.2023.1119547

**Published:** 2023-03-03

**Authors:** Thelma Ameh, Kuzy Zarzosa, W. Evan Braswell, Christie M. Sayes

**Affiliations:** ^1^ Department of Environmental Science, Baylor University, Waco, TX, United States; ^2^ Insect Management and Molecular Diagnostics Laboratory, USDA APHIS PPQ S&T, Edinburg, TX, United States

**Keywords:** silver nanoparticles, copper nanoparticles, nanoparticle surface, antibacterial action, read across

## Abstract

Nanoparticles have been proposed as tunable delivery vehicles for targeted treatments and, in some cases, the active therapeutic agents themselves. Despite the promise of such customizable impacts, little evidence exists to support these claims in the realm of antibiotics. Exploration of the silver and copper nanoparticle antibacterial impacts have been reported with inconsistent results. Here, we investigate the physical, chemical, and bacterial properties of silver and copper core particles stabilized with commonly used surface coatings, namely, polyvinylpyrrolidone (PVP, to confer a neutrally charged surface), cetrimonium bromide (CTAB, positively charged surface), citrate (Cit, negatively charged surface for silver nanoparticles), and ascorbic acid (AA, negatively charged surface for copper nanoparticles. The impacts of these potential antibacterial nanoparticles are measured against three bacterial species spanning deep divisions in the bacterial tree of life and include *Escherichia coli*, *Staphylococcus aureus*, and *Sphingobacterium multivorum*. Varying dose, core composition, surface coating, and bacterial species revealed that nanoparticle surfaces accounted for most of the variation in antibacterial activity. In all experiments, dose produced a linear inhibitory effect. Surprisingly, bacterial species reacted similarly regardless of evolutionary relatedness. There is a high degree of consistency, effectiveness, and efficacy among PVP silver and copper nanoparticle. These findings have implications for the intentional use of nanotechnology in environmental systems.

## Introduction

Targeted vehicles are needed to chaperone therapeutics through biological systems as diverse as humans and plants for the treatment of disease. Nanoparticles can serve as a customizable platform for the delivery of payloads and in some cases, the particles themselves can also function as the active therapeutic agent (Sahoo and Labhasetwar, 2003; Singh and Lillard, 2009; Sayes et al., 2017). For example, nanoparticles that have shown antibacterial properties are postulated as novel drug carrier systems that induce the advantageous side-effect of killing harmful bacteria (Tian et al., 2007; Bhattacharya and Mukherjee, 2008; AshaRani et al., 2009; Prow et al., 2011; Pelgrift and Friedman, 2013). The implications for the intentional use of nanotechnology in agricultural and other environmental systems requires systematic investigations where physical, chemical, and biological properties are varied in controlled studies.

An understanding of the interactions between nanoparticles and bacteria is required to characterize the antibiotic effects. Because of the particle’s size, the interaction often occurs with the cell wall of bacteria. The interaction is dynamic involving the surface of the nanoparticle with distinct properties and the fluidic nature of the cell’s surface made of lipids, receptors, and/or cellulose (Nel et al., 2009). The interaction produces direct effects, such as particle uptake and subsequent accumulation in lipid bilayers or vesicles (Pagano and Weinstein, 1978; Berg et al., 2010; Mu et al., 2012). Nanoparticle exposure can also produce indirect effects, such as the production of reactive oxygen species (ROS) and subsequent oxidative stress (Ševců et al., 2009; von Moos and Slaveykova, 2014; Kumar et al., 2011; Premanathan et al., 2011; Li et al., 2012).

The charged cell surface of bacteria readily attracts charged nanoparticle surfaces *via* electrostatics (Li et al., 2011; Sadiq et al., 2011; Ma and Lin, 2013). The attraction is followed by adsorption; then a series of adverse outcomes are induced, such as cell wall disruption, intracellular leakage, or disrupted metabolism (Nel et al., 2006; Wigginton et al., 2010; Cherchi et al., 2011; Kumar et al., 2011; Dalai et al., 2012; Ma and Lin, 2013). The surface charge of nanoparticles (conferred by the surface coating) plays a role in their antimicrobial properties. Nanoparticle synthesis typically concludes with the addition of a stabilizing agent to halt particle growth (Brust et al., 1994). This surface property has been shown to alter uptake and cytotoxicity effects in mammalian cells (Berg et al., 2009; Asati et al., 2010; Mahmoud et al., 2010; Fröhlich, 2012). But, to-date, there is no report available that analyzed the effects of nanoparticle surface coatings among different bacteria species.

The study summarized in this paper investigated the antibacterial properties of silver and copper particles stabilized with citrate (Cit) and ascorbic acid (AA), respectively, to confer a negatively charged surface; polyvinylpyrrolidone (PVP) to confer a neutrally charged surface; and cetyltrimethylammonium bromide (CTAB) to confer a positively charged surface. Three bacterial species representing deep divisions in the bacterial tree of life were used for endpoint analysis: *Escherichia coli* (Proteobacteria), *Staphylococcus aureus* (Firmicutes), and *Sphingobacterium multivorum* (Bacteroidota). The findings show that nanoparticles can be customized for diverse antibacterial strategies.

## Materials and methods

*Reagents.* Trisodium citrate (Na_3_C_6_H_5_O_7_, CAS# 6132-04-3, 1% w/v) and polyvinylpyrrolidone (PVP, CAS# 9003-39-8, MW 40,000) were purchased from Alfa Aesar (Haverhill, Massachusetts, United States) and silver nitrate (AgNO_3_, CAS# 7761-88-8, >99.9%) was purchased from Ricca Chemical Company (Arlington, Texas, United States). Sodium hydroxide (NaOH, CAS# 1310-73), copper (II) nitrate trihydrate (Cu(NO_3_)_2_·3H_2_O, CAS# 10031-43-3), ethanol (EtOH, C_2_H_5_OH, CAS# 64-17-5), and cetyl trimethylammonium bromide (CTAB, C_19_H_42_BrN, CAS# 57-09-0) were purchased from Sigma-Aldrich (St. Louis, Missouri, United States). L-ascorbic acid (C_6_H_8_O_6_, CAS# 50-81-7), copper (II) chloride (CuCl_2_, CAS# 7447-39-4) and hydrazine hydrate (H_6_N_2_O, CAS# 7803-57-8) were purchased from Acros Organics (Thermo Fisher Scientific, Waltham, Massachusetts, United States). Mueller Hinton agar was purchased from Oxoid Ltd. (Cheshire, England), while Mueller Hinton broth, Nutrient agar, Nutrient broth and 0.5 McFarland standard were purchased from Remel (Thermo Fisher Scientific). Bacterial culture *Escherichia coli* (*E. coli*) 25992 and *Staphylococcus aureus* (*S. aureus*) 6538 were purchased from Microbiologics (St. Cloud, Minnesota, United States). *Sphigobacterium. multivorum* (*S. multivorum*) 5,011 was collected from Mexican fruit fly colonies.

*Nanoparticle synthesis.* Citrate coated silver nanoparticles were synthesized *via* trisodium citrate (10 mL of 1%) added dropwise to silver nitrate (500 mL of 1 mM) under vigorous magnetic stirring for 1 h at 150°C until a grey-colored particle suspension, termed ‘Cit-AgNPs’, was observed (Pillai and Kamat, 2004; Ledwith et al., 2007; He et al., 2013). Similarly, polyvinylpyrrolidone coated silver nanoparticles were synthesized *via* silver nitrate (2 mL of 5%) added dropwise to PVP (100 mL of 2%) under vigorous magnetic stirring at 100°C (Wang et al., 2005; Samadi et al., 2010). The reaction occurred in the dark for 1 hour until a green-colored particle suspension, termed ‘PVP-AgNPs’, was observed.

Cetyltrimethylammonium bromide coated silver nanoparticles were synthesized *via* silver nitrate (50 mL of 0.01 M) added dropwise into CTAB (50 mL of 0.01 M) under vigorous magnetic stirring until a milky white opalescent suspension slowly formed. In a separate reaction vessel, sodium hydroxide (50 mL of 0.01 M) was added to glucose (25 mL of 5.0 mM) under vigorous magnetic stirring (Khan et al., 2009). Solutions were combined and stirred while heated at 50°C for 5 h until the formation of an amber-colored particle suspension, termed ‘CTAB-AgNPs’.

Ascorbic acid coated copper nanoparticles were synthesized *via* ascorbic acid (250 mL of 1.0 M) added dropwise to copper chloride (250 mL of 10 mM) while being stirred vigorously at 80°C for 17 h until the formation of brown-colored particle suspension, termed ‘AA-CuNPs’ was observed (Wu et al., 2005; Wu et al., 2006). Similarly, polyvinylpyrrolidone coated copper nanoparticles were synthesized *via* 1:1 ratio by volume of freshly prepared PVP (0.8 M) and copper (II) nitrate trihydrate (0.01 M) and stirred at 45°C for 3 h until the formation of pink-colored particle suspension, termed ‘PVP-CuNPs’ (Wu et al., 2006).

Cetyltrimethylammonium bromide coated copper nanoparticles were synthesized *via* 1:1 ratio by volume of copper (II) chloride (1.0 mM; pH adjusted to 10) and CTAB (0.01 M) mixed with hydrazine hydrate (0.08) (Chen et al., 2006). Solution was mixed vigorously for 3 h until the formation of crimson-colored particle suspension, termed ‘CTAB-CuNPs’, was observed. All resultant nanoparticle suspensions were centrifuged to remove unreacted materials.

*Nanoparticle spectroscopic characteristics.* Optical absorption spectra of the synthesized nanoparticles were examined using ultraviolet/visible spectroscopy (UV/Vis; Agilent 8,453; Shangai, China) to confirm the presence of nanoparticle colloids. The surface coating was confirmed *via* Fourier-transform infrared spectrophotometry (FTIR; Nicolet iS10, Thermo Scientific). Nanoparticle surface charge was assessed using zeta potential measurements collected *via* dynamic light scattering (DLS; ZEN 3690 Nanoseries Zetasizer; Malvern, Worcestershire, UK).

In addition to spectroscopy, morphological characteristics were observed using microscopy. Transmission electron microscopy (TEM) was performed on JEOL JEM-1010 TEM (Tokyo, Japan) at an accelerating voltage of between 10 and 100 kV. Briefly, a drop of the prepared particle suspension was added to the surface of carbon-coated copper grids (Electron Microscopy Sciences; Hartfield, Pennsylvania, United States) and allowed to dry in a hot air oven set at 160°C. Atomic force microscopy (AFM) was performed using Bruker Dimension Icon Atomic Force Microscope (Santa Barbara, California, United States) in tapping mode.

*Antibacterial assay.* The antibacterial efficiency of the nanoparticles synthesized were tested against *E. coli*, *S. aureus*, and *S. multivorum* using the agar disc diffusion method (Bonev et al., 2008). For each nanoparticle, the experiment was carried out in triplicate using plates containing three discs per plate. The nanoparticle-loaded discs were prepared by impregnating 7 mm plain discs with each particle suspension at four dosing concentrations (1, 2, 4, and 8 nM). Each suspension (5 µL) was pipetted onto each disc and dried in an oven at 80°C for 2 h. For the propagation of *E. coli* and *S. aureus*, a broth inoculum was made by suspending a pure bacteria colony from an 18-24 h Mueller Hinton agar culture plate into a Mueller Hinton broth tube. The broth tube was then incubated at 37°C for 6 h until turbidity of a 0.5 McFarland standard was achieved. Mueller Hinton agar plates were then inoculated by spreading swab-steeped bacteria from the broth tube over the entire sterile agar surface. Colonies of *S. multivorum* were propagated from an 18-24 h Nutrient agar plate and transferred to nutrient broth tubes incubated at 32°C for 6 h after which the turbidity was adjusted to a 0.5 McFarland standard. Nutrient agar plates were then inoculated by spreading swab-steeped bacteria from the broth tube over the entire sterile agar surface. Flame and ethanol sterilized forceps were used to place three particle-impregnated discs on the surface of the culture plates. The resultant plates for *E. coli* and *S. aureus* were incubated at 37°C for 18 h, while *S. multivorum* plates were incubated at 32°C for 18 h.

After incubation, the zone of bacteria growth inhibition around each disc was measured to the nearest millimeter. Zone of inhibition is traditionally measured as the diameter of the zone defined by no bacterial growth on a bacterial lawn. That is, the measurement is taken from the edge of bacterial growth on one side through the filter paper disk to the edge of bacterial growth on the other side. Zero inhibition is indicated by bacterial growth directly at the edge of a filter paper disc soaked in antibacterial agent being tested. Because the zone of inhibition includes the diameter of the filter paper disc, this classification system results in a distribution of inhibitory diameters that is not continuous. That is, the smallest value is zero and the second smallest value is larger than the disc size with no possible values between those two points. To better fit a continuous distribution, we subtracted the diameter of the filter paper disc from the diameter of the zone of inhibition measurements. Furthermore, because the data were bounded by zero and positively skewed, we used a square root transformation (*i.e.,* the square root of each measure of inhibition was used for further analysis) to approximate a normal distribution. Values were transformed for meaningful presentation.

*Statistical analysis.* To assess the impact of the treatments on bacterial inhibition, we used factorial analysis of variance (*i.e.,* ANOVA) to fit a model that includes the following independent factors: nanoparticle core, surface coating, dose, and species. Comparison of means between levels of each factor was performed using Tukey HSD test (*α* = 0.05). To estimate the amount of effect of each independent factor (*i.e.,* the proportion of variation explained by each factor), we calculated eta-squared, η^2^, using the formula

$$\eta^{2}=\frac{{SS}_{effect}}{{SS}_{total}}$$

which indicates large effects at 0.14, medium at 0.06, and small effect sizes at 0.01 (Cohen, 1988). All statistical tests were performed in JMP^©^, Version 13 (SAS Institute Inc, Cary, NC, 2016).

## Results

**FIGURE 1:**
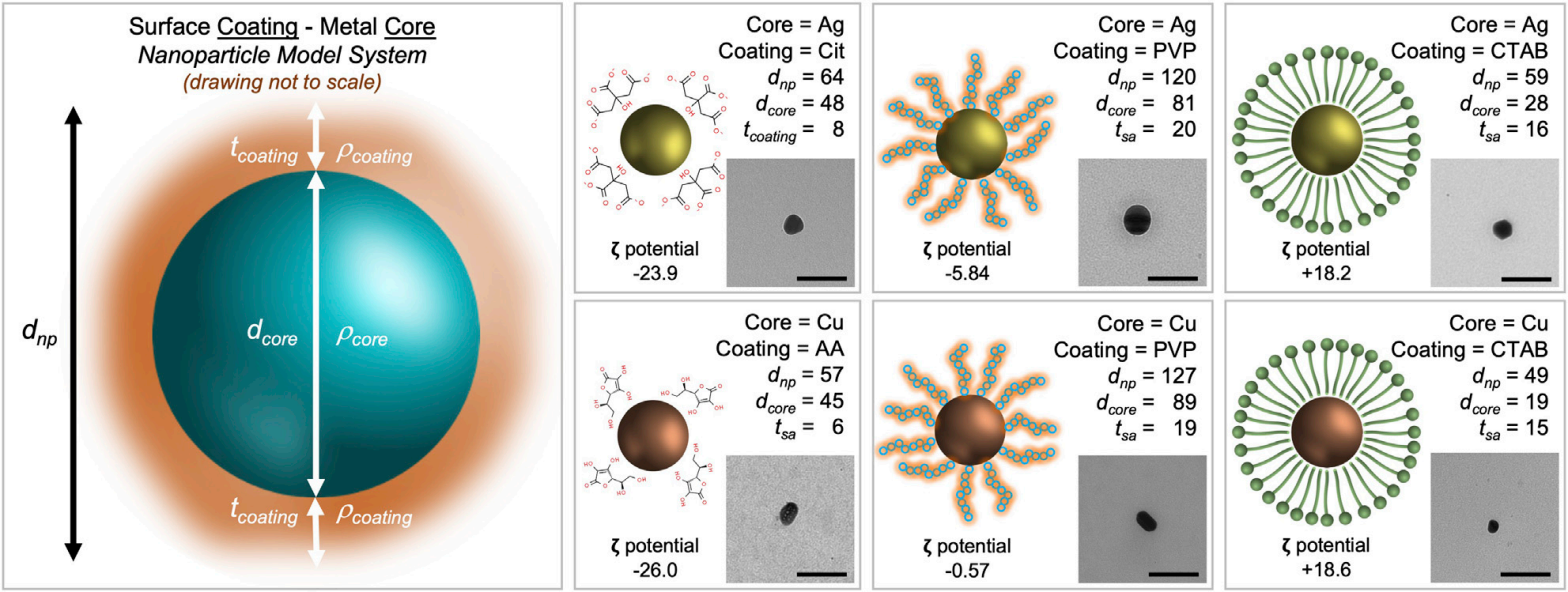
Morphological characterization scheme for metal nanoparticles with stabilizing agents. The core density ( $\rho_{core}$ ) and surface coating density ( $\rho_{coating}$ ) are assumed the same as the bulk density of the metal composition (silver (Ag) $\rho_{core}$ = 10.49 g/cm³ and copper (Cu) *ρ*_*core*_ = 8.96 g/cm³) and surface coating (citrate (Cit) *ρ*_*coating*_ = 1.66 g/cm³; ascorbic acid (AA) *ρ*_*coating*_ = 1.65 g/cm³; polyvinylpyrrolidone (PVP) *ρ*_*coating*_ = 1.25 g/cm³; cetyltrimethylammonium bromide (CTAB) *ρ*_*coating*_ = 1.33 g/cm³). Transmission electron microscopy (TEM) is the method used to assess core diameter (*d*_*core*_); this technique does not provide sufficient information about the surface coating (due to the low contrast of organic material). Fourier transform infrared (FTIR) spectroscopy is used to provide evidence of nanoparticle associated surface coating (Supplementary Figure S1). The diameter of the nanoparticle core is measured relative to the scale bar (100 nm) and is reported as the average of more than 500 particles. To measure total nanoparticle diameter (*d*_*np*_), dynamic light scattering (DLS) is used. By subtracting the diameter of the core (*d*_*core*_ as measured by TEM) from the hydrodynamic diameter of the total particle system (*d*_*np*_) and dividing by 2, the thickness of the surface coating is estimated ( $t_{shell}=\frac{d_{np-}d_{core}}{2}$ ). Atomic force microscopy (AFM) is used to verify TEM and DLS measurements (Supplementary Figure S1). No single technique can measure all the morphological parameters of a nanoparticle system with a single experiment.

Reporting physical and chemical properties aids in the identification of additional descriptors that are important for comparison among previously reported studies as well as enabling read across for comparative analyses. We have included microscopic and spectroscopic data of the nanoparticles that were used in this study. Transmission electron microscopy (TEM) confirmed particle dimensions in the x-y plane and indicates the nanoparticle’s propensity to agglomerate (Figure 1 **)**. Atomic force microscopy (AFM), a type of scanning probe microscopy, has a demonstrated resolution on the order of fractions of a nanometer and was used to measure the height (*z*-axis) of each nanoparticle suspension (Supplementary Figure S1). Dynamic light scattering (DLS) of each nanoparticle suspension was measured to estimate zeta potential and hydrodynamic diameter (a measure of particle size).

When inferring particle size from both TEM (to measure the core) and DLS (to measure the entire nanoparticle system), the size data shows trends. Cit-AgNP and AA-CuNPs have a *d*_*np*_ of 64 nm (*d*_*core*_ of 48 nm) and 57 nm (*d*_*core*_ of 45), respectively. The thickness of the citrate coating is 8 nm while the thickness of the ascorbic acid coating is 6 nm. PVP-AgNP and PVP-CuNPs have a *d*_*np*_ of 120 nm (*d*_*core*_ of 81 nm) and 127 nm (*d*_*core*_ of 89n m), respectively. The thickness of the PVP coating ranges 19–20 nm. CTAB-AgNP and CTAB-CuNPs have a *d*_*np*_ of 59 nm (*d*_*core*_ of 28 nm) and 49 nm (*d*_*core*_ of 19 nm), respectively. The thickness of the CTAB coating ranges 15–16 nm. All nanoparticles appear spheroidal in shape. Zeta potential data show that Cit-AgNPs and AA-CuNPs have negatively charged surfaces (−23.9 mV vs. −26.0 mV), while PVP-AgNPs and PVP-CuNPs have values close to 0 (−5.84 mV vs. −0.57 mV). Zeta potential data show that CTAB-AgNPs and CTAB-CuNPs have positively charged surfaces (+18.2 mV vs. +18.6 mV).

Ultraviolet-visible (UV-Vis) spectroscopy was used to confirm the presence of silver and copper nanoparticles in the suspensions (data not shown). All six nanoparticle suspensions produced a single peak indicating one distinct size population per sample. The UV-Vis absorbance peaks of the nanoparticles were observed between 300 and 450 nm (Aoki et al., 2003; Percival et al., 2007). The absorbance peak between 400 and 450 nm is indicative of AgNP formation and the peak between 300 and 400 nm is indicative of CuNP formation (Evanoff and Chumanov, 2004; Peng et al., 2010; Patil et al., 2018).

Fourier-transform infrared spectroscopy (FTIR) was used to verify the surface coating by identifying chemical composition (Kumar et al., 2000; Zhang et al., 2007; Chen et al., 2008). This information provides a glimpse into the reducing property of the stabilizing agent after nanoparticle synthesis. At each wavenumber position, the fluctuation corresponds to a bending or stretching frequency of a particle bond. The red or blue shift of the wavenumber position indicates the close association of a C=O group of the PVP or CTAB or an -O group of the citrate or ascorbic acid to the silver or copper particle surface, which is often cited as a stable association between a nanoparticle core and the surface coating.

Three bacterial species representing deep divisions in the bacterial tree of life were used for endpoint analysis: *Escherichia coli* (Proteobacteria), *Staphylococcus aureus* (Firmicutes), and *Sphingobacterium multivorum* (Bacteroidota). *E. coli* is Gram-negative bacterium and is a common human bacterial pathogen. *S. aureus* is a Gram-positive bacterium and is the leading cause of soft tissue infection. *S. multivorum* is a Gram-negative bacterium and grows in antiseptics and disinfectants. Each of these bacteria species were used in the Kirby-Bauer disk diffusion susceptibility test.

The disk diffusion susceptibility method tests the effectiveness of antibiotics on a specific microorganism (Jorgensen and Ferraro, 2009). The zone of inhibition is a circular area around the spot of the antibiotic in which the bacteria colonies do not grow (Joshi et al., 2011). Bacterial cell membrane disruption is the most commonly cited cytotoxicity mechanism induced after engineered nanoparticle exposure (McShan et al., 2014). The most effective antibacterial engineered nanoparticles are metal-containing particles in the size range of 20–90 nm. However, nanoparticles have many more inherent physicochemical characteristics; and how these other factors contribute to antibacterial properties are not well understood.

**FIGURE 2:**
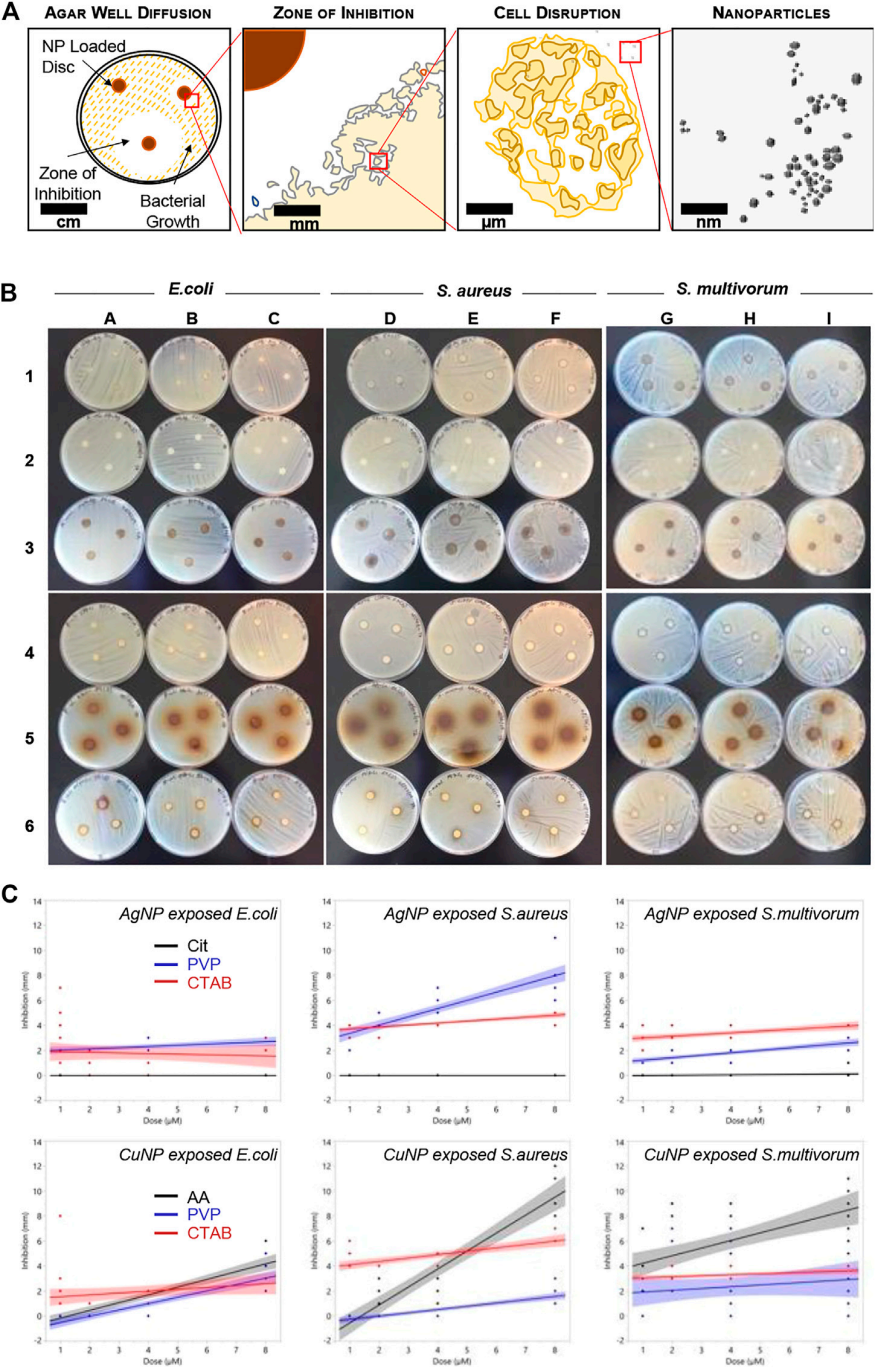
Bacterial growth inhibition resulting from nanoparticle exposure. **(A)** Schematic representing the disc diffusion method analyses. **(B)** Photographs of the disc diffusion results. **(C)** Quantitative representation of the photographs. Plots show inhibition (measured in mm) induced in *Escherichia coli*, *S. aureus*, and *S. multivorum*. Blue dots indicate measures of inhibition from Cit-AgNPs (top row) and AA-CuNPs (bottom row), red dots indicate measures from PVP-AgNPs and PVP-CuNPs, and black dots indicate measures from CTAB-AgNPs and CTAB-CuNPs. Lines indicate the best fit regression and shaded areas represent the 95% confidence interval.

**FIGURE 3:**
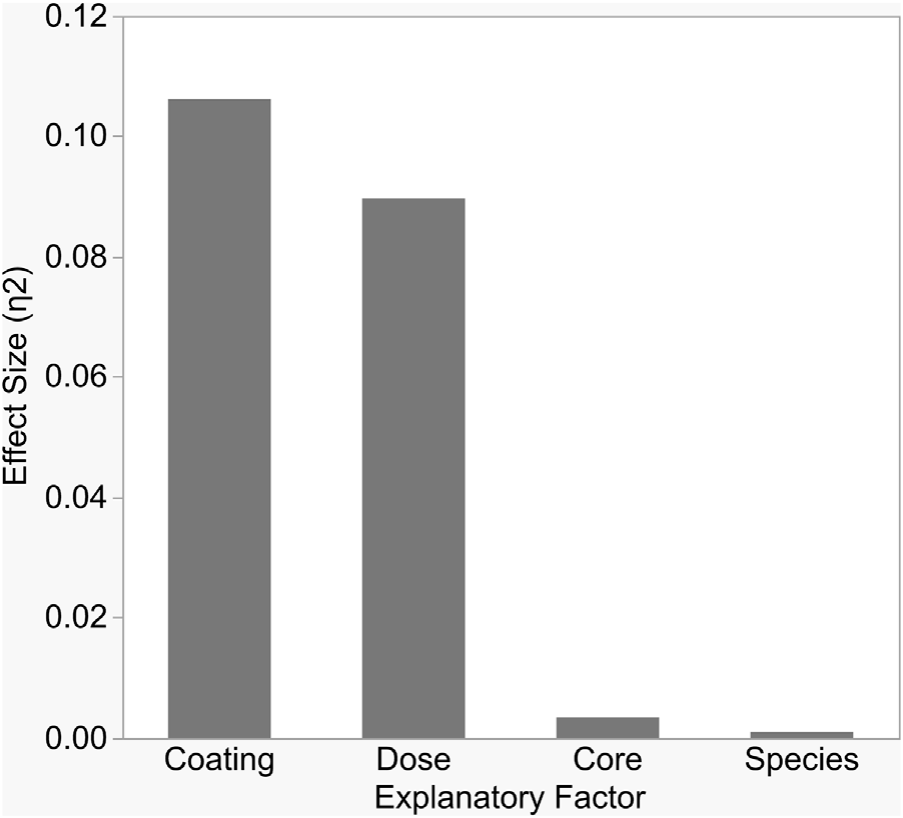
Each variable in the study significantly impacted bacterial growth inhibition, but the size of impact differed between variables. As shown here, surface coating and dose explained the largest proportions of variation in inhibition, as measured by partial Eta-squared.

Antibacterial sensitivity tests showed that Cit-AgNPs did not inhibit growth of *E. coli*, *S. aureus,* or *S. multivorum* at any concentration tested (Figure 2; Table 1). All other particles tested showed a dose-dependent inhibition against the growth of *E. coli*, *S. aureus,* and *S. multivorum*. The impact of core, surface coating, dose, and species (as well as all possible interactions) were assessed on the size of the bacterial zone of inhibition. There was significant variation between groups; the statistical model explained a large proportion of variation in bacterial inhibition (F_(71, 576)_ = 97.31, *p* < 0.0001, R^2^_adj._ = 0.91) (Figure 3). Each factor, as well as all interactions among factors, explained a significant portion of the variation in bacterial inhibition (*p* < 0.00001, except species where *p* < 0.03). However, the proportion of variation explained by each factor, that is, the size of the effect (*i.e.,* η^2^), differed greatly. Overall, surface coating had the largest effect on bacterial inhibition (F_(2, 576)_ = 397.28, *p* < 0.0001, η^2^ = 0.11).

**TABLE 1:** Summary of regression analyses of dose and inhibition (Figure 2C) for each species and each nanoparticle. The table provides values indicating antibacterial efficacy per surface coating that may be used for two different types of strategies. For one strategy, if complete inhibition is required, the selection of CTAB-AgNPs would be ideal. For a second strategy, when increased inhibition over increasing dose is desired, then PVP-AgNPs, AA-CuNPs, PVP-CuNPs, or CTAB-CuNPs are ideal. On the other hand, Cit-AgNPs are ineffective in antibacterial efficacy based on the experiments used in these systematic studies.

Nanoparticle exposure	Species	Dose-response relationship (β)	F_(1,34)_	Coefficient of determination (R^2^)	*p*-Value
Cit-AgNPs	*E. coli*	0	NA	NA	NA
Cit-AgNPs	*S. aureus*	0	NA	NA	NA
Cit-AgNPs	*S. multivorum*	0.02	2.63	0.07	0.1141
PVP-AgNPs	*E. coli*	0.09	7.23	0.18	0.0110
PVP-AgNPs	*S. aureus*	0.66	116.99	0.77	<0.0001
PVP-AgNPs	*S. multivorum*	0.20	49.80	0.59	<0.0001
CTAB-AgNPs	*E. coli*	−0.05	0.29	0.01	0.5942
CTAB-AgNPs	*S. aureus*	0.16	52.63	0.61	<0.0001
CTAB-AgNPs	*S. multivorum*	0.14	17.18	0.34	0.0002
AA-CuNPs	*E. coli*	0.62	135.25	0.80	<0.0001
AA-CuNPs	*S. aureus*	1.44	171.52	0.84	<0.0001
AA-CuNPs	*S. multivorum*	0.61	23.93	0.41	<0.0001
PVP-CuNPs	*E. coli*	0.51	118.66	0.78	<0.0001
PVP-CuNPs	*S. aureus*	0.26	86.57	0.72	<0.0001
PVP-CuNPs	*S. multivorum*	0.14	1.08	0.03	0.3062
CTAB-CuNPs	*E. coli*	0.15	3.24	0.09	0.0809
CTAB-CuNPs	*S. aureus*	0.27	36.71	0.52	<0.0001
CTAB-CuNPs	*S. multivorum*	0.08	9.86	0.23	0.0035

Tukey’s HSD (honestly significant difference) test identified significant differences between the three different nanoparticle surface coatings with bacterial growth inhibition increasing with zeta potential values (least squares mean ± SD: Cit/AA = 0.12 ± 0.001 mm; PVP = 0.49 ± 0.001 mm; CTAB = 3.2 ± 0.001 mm). Dose explained a moderate proportion of variation in bacterial inhibition with (F_(3, 576)_ = 223.64, *p* < 0.0001, η^2^ = 0.09). Significantly greater inhibition occurred at the 8 nM dose (least squares mean ± SD: 8 nM = 2.87 ± 0.0005 mm) and significantly reduced inhibition occurred at the 1 nM dose (least squares mean ± SD: 1 nM = 0.89 ± 0.0005 mm). However, the 2 and 4 nM doses did not differ from each other (least squares mean ± SE: 2 nM = 1.2 ± 0.0005 mm; 4 nM = 1.35 ± 0.0005 mm). While statistically significant, core and species alone had only a slight effect on inhibition (F_(1, 576)_ = 25.71, *p* < 0.0001, η^2^ = 0.003 and F_(2, 576)_ = 3.76, *p* < 0.02, η^2^ = 0.001, respectively).

The major contributors to bacterial inhibition in this study are highly dependent upon the surface coating and dose. Dose produced a linear effect. The effect of surface coating, however, is mediated by the core. The interaction between these two variables leads to the conclusion that species react similarly regardless of evolutionary relatedness. Thus, general statements can be made about similarities and differences in nanoparticle antibacterial properties.

**FIGURE 4:**
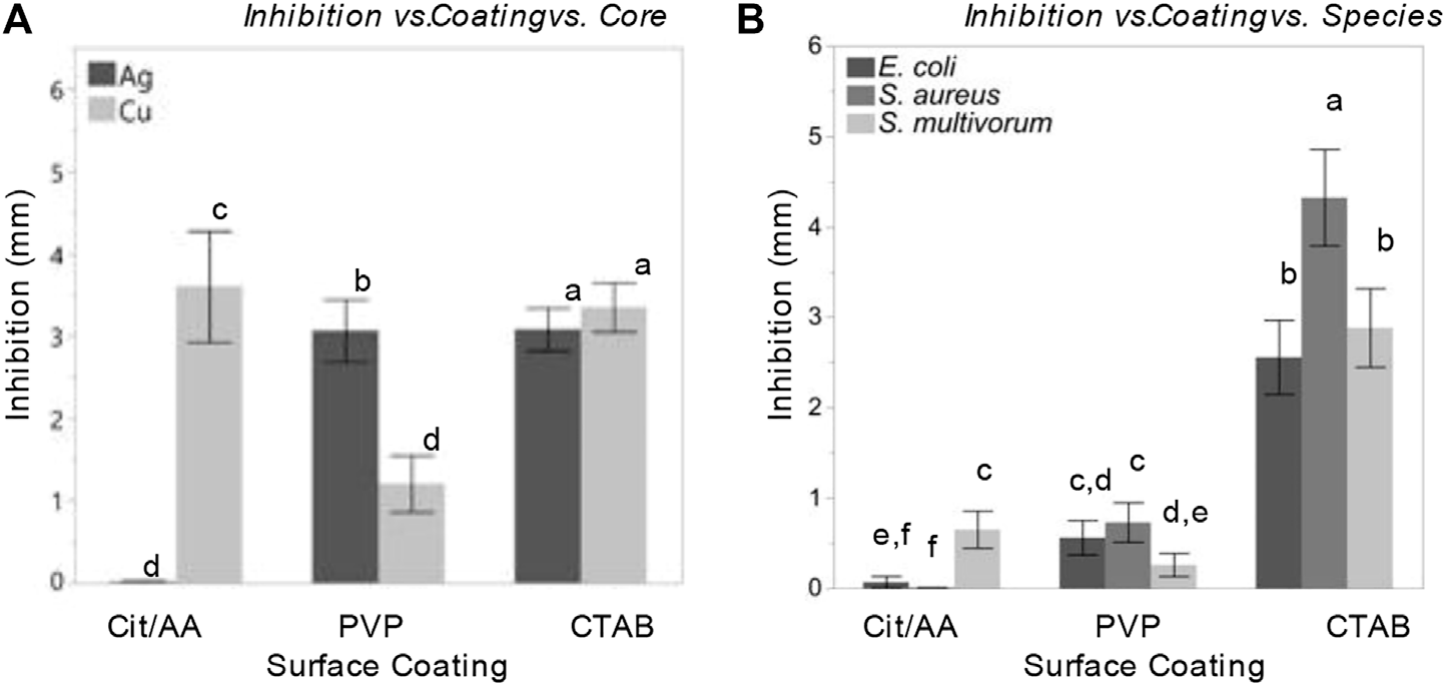
Nanoparticles and their surface coatings produce distinct antibacterial effects but have consistent impact across diverse bacterial species. Comparison of least squares means and 95% confidence intervals for bacterial inhibition following nanoparticle treatment resulting from a model accounting for **(A)** inhibition effects as a function surface coating and core; **(B)** inhibition effects as a function surface coating and species.

Interactions among the main factors were statistically significant (Figure 4). Core influenced the relationship between surface coating and inhibition (F_(2, 576)_ = 201.01, *p* < 0.0001, η^2^ = 0.05). Cit-AgNPs induced almost no inhibition in bacterial growth while PVP-AgNPs and CTAB-AgNPs induced significantly greater levels of inhibition (least squares mean ± SD: Cit = 2.5 ± 0.003 mm; PVP = 1.94 ± 0.003 mm; CTAB = 3.14 ± 0.003 mm). All CuNPs, on the other hand, induced bacterial inhibition, but PVP-CuNPs induced significantly less inhibition than AA-CuNPs and CTAB-CuNPs (least squares mean ± SD: PVP = 0.49 ± 0.003 mm; AA = 4.93 ± 0.003 mm; CTAB = 3.26 ± 0.003 mm).

**FIGURE 5:**
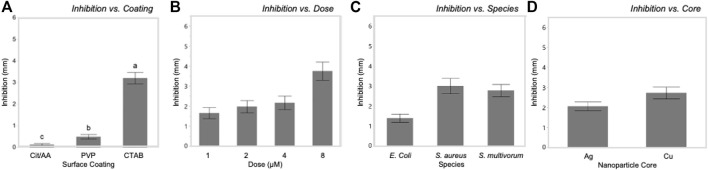
Bacterial growth inhibition was significantly influenced by each of the main factors in this systematic study. Inhibition increased with increasing zeta potential of surface coatings **(A)** and dosage **(B)**. Inhibition varied among bacterial species **(C)** and between nanoparticle cores **(D)**.

Dose influenced the relationships between surface coating and core with inhibition. In *E. coli*, PVP-AgNPs showed a relatively constant zone of inhibition over the dosing concentrations (1, 2, 4, and 8 nM), while the zone of inhibition within CTAB-AgNP treatment exhibited the greatest inhibition at the lowest concentration tested. The zones of inhibition within all CuNP treatments where greatest at the highest concentration. Most notably, the mean zone of inhibition within AA-CuNP treatment is 4.53 mm at 8 nM and within PVP-CuNP treatment is 3.39 mm at 8 nM, which are some of the largest inhibition zone mean values observed in this study. Figure 5 shows comparisons of the variables used in the study.

All particles tested except for Cit-AgNPs showed a dose-dependent inhibition against the growth of *S. aureus*. PVP-AgNPs zone of inhibition increased gradually from 2.88 to 7.61 mm as the concentration increased. CTAB-AgNPs zone of inhibition slightly increased from 3.88 to 4.88 mm when the concentration reached 8 nM. Cit-CuNPs showed no growth inhibition against *S. aureus* at the lowest concentration tested, with little growth inhibition at 2 and 4 nM concentrations (1.42–1.49 mm), but this drastically increased to 10.23 mm at 8 nM concentration.

The antibacterial action of PVP-AgNPs and CTAB-AgNPs against the growth of *S. multivorum*, were also relatively constant, ranging 1.0–2.53 mm and 2.74–4.0 mm over the dosing concentrations (1, 2, 4, and 8 nM), increasing only slightly as concentration increased. Bacterial inhibition by CTAB-CuNPs and AA-CuNP were dose-dependent with the highest zones of inhibition (3.54 and 8.15 mm, respectively) produced at the highest concentration. No growth inhibition was observed by PVP-CuNPs at the lowest concentration; however, a mean zone of inhibition of 3.4 mm was observed at 2 nM, with decreasing growth inhibition as concentration increased. Most notably, the mean zone of inhibition after AA-CuNP treatment was 8.15 mm at 8 nM and after PVP-AgNP treatment is 2.53 mm at 8 nM.

## Discussion

Nanoparticle-induced inhibition of bacterial growth can be thought of from three different perspectives: the influence of the surface coating, the core, and the species. When reviewing the effects of surface coating on inhibition, overall CTAB performed the best. CTAB-AgNPs and CTAB-CuNPs induced inhibition (at 1 nM) and showed a consistent dose-response (up to 8 nM) in all bacteria species tested. PVP also performed well in that a high degree of consistency, effectiveness, and efficacy was observed between PVP-AgNPs and PVP-CuNPs. These nanoparticle systems were mostly inhibitory (at 1 nM) and showed a gradual dose-response relationship (up to 8 nM).

In terms of core, the suite of copper nanoparticles produced greater zones of inhibition as compared to the suite of silver nanoparticles. AA-CuNPs produced the highest zones of inhibition among all nanoparticle systems and all bacteria species used in the study. Table 1 summarizes the observations related to surface coating and core on inhibition and dose-response.

Each nanoparticle system induced remarkably similar patterns of responses in all three bacterial species. While species identity did influence the degree of inhibition, it did so mainly through interactions with other variables and the size of this effect was substantially smaller than the effect of surface coating or dose. Moreover, the peptidoglycan layer of Gram-positive bacteria has been hypothesized to influence the impact of nanoparticles on bacterial growth (Hemeg, 2017; Zaidi et al., 2017; Lee et al., 2019). However, the degree of inhibition was more similar between the Gram-positive and Gram-negative species (*S. aureus* and *S. multivorum*) than between the two Gram-negative species (*E. coli* and *S. multivorum*).

Ultimately, the similarity in species’ responses suggests that the interactions between nanoparticles and bacteria impact cellular structures, or alter cellular processes, that are highly conserved across the bacterial tree of life. Further exploration should focus on similarities of response among more diverged branches of the tree of life (*i.e.,* animals, plants, fungi) as well as on other nanoparticle organic (or inorganic) surface coatings that may induce inhibition. The variable that explained most variation in bacterial growth inhibition was nanoparticle surface coating. This result provides guidance on the design of nanoparticles with specific antibiotic results in mind. Citrate coated silver nanoparticles might be chosen as a drug delivery vehicle when preservation of bacterial communities is needed. Alternatively, when bacterial inhibition is the goal, CTAB coated copper nanoparticles would be preferred. This critical finding offers a path forward for the long-promised goal of designer nanoparticles.

Previous studies have demonstrated antibacterial properties of silver and copper in a variety of different formulations (Lin et al., 1998; Chen et al., 2006; Brunel et al., 2013). Results suggest the high surface area to volume ratio of nanoparticles in comparison to bulk materials make them more toxic to microorganisms (Willey et al., 2008; Ma et al., 2012; Sadeghi et al., 2012; Bondarenko et al., 2013; McQuillan and Shaw, 2014; Pareek et al., 2018). Nanoparticle size influences dissolution rate, surface coating impacts the release of ions from nanoparticles (Damm and Münstedt, 2008; Kittler et al., 2010; Pareek et al., 2018) while shape enhances the antimicrobial activity (Pal et al., 2007; Sadeghi et al., 2012). Results as they relate to nanoparticle core have been inconsistent. We conclude that core has a relatively minor impact on bacteria inhibition. Considering these results, we hypothesize that the discrepancies in the literature may be explained by variation in the nanoparticle surface coating used in each study.

## Conclusion

The patterns that emerged among the six nanoparticle systems and the three bacteria species are unique between the metal core and the surface coating but consistent among bacterial species. Each species responded similarly to each surface coating independent of the metal core. Given the distant evolutionary relationships between these species, we hypothesize that these molecules impact anciently evolved cellular pathways and, thus, predict that these effects may be universal among bacteria. Such ancient traits may be subject to strong purifying selection and thus resistant to selection imposed by antimicrobials The emergence of patterns such as these demonstrate the utility of systematically investigating the antibacterial properties of nanoparticles.

The findings have implications for the intentional use of engineered nanoparticles in environmental systems. Importantly, we have demonstrated that the tunability of nanoparticle-based antibacterial agents are primarily dependent on the surface features of the particle system. This may be because it is the surface of the particle that interacts with the bacteria’s cell membrane; therefore, surface interactions ought to be the focus of any design of new antibacterial materials. Of note, however, is that the data presented here reflect immediate and short-term interactions. Yet, nanoparticles are likely to degrade through time. Work is needed to explore the impacts of degradation and the timeline over which the metal core may become more important. This information will be critical to developing time-released formulations and understanding the ecological and environmental impacts of nanoparticle-based therapeutics.

## Data Availability

The raw data supporting the conclusion of this article will be made available by the authors, without undue reservation.
