# Supplementary material for: Nanoparticle surface coatings produce distinct antibacterial effects that are consistent across diverse bacterial species
Source: Front Toxicol. 2023 Mar 3;5:1119547. doi: 10.3389/ftox.2023.1119547 (PMC10022730; doi:10.3389/ftox.2023.1119547)
Supplement: Supplementary file 1 [file DataSheet1.docx]

*For submission to Frontiers in Toxicology*

**SUPPLEMENTAL INFORMATION:**

**Customizable nanoparticles for diverse antibacterial strategies**

Thelma Ameh1,  Kuzy Zarzosa2,  W E. Braswell2 and  Christie M. Sayes1*

**Affiliation:**

^1^ Department of Environmental Science, Baylor University, Waco, TX 76798

^2^ Mission Laboratory, United States Department of Agriculture, Animal and Plant Health Inspection Service, Plant Protection and Quarantine, Science and Technology, Edinburg, TX 78541

^*^ **Corresponding Authors:** Evan Braswell; Address: Moore Air Base, Bldg 6414, 22675 N. Moorefield Rd., Edinburg, TX 78541; Phone: (956) 205-7660; Email: evan.braswell@usda.gov

Christie Sayes; Address: One Bear Place #97266, Waco, TX 76798-7266; Phone: (254) 710-3469; Email: christie_sayes@baylor.edu

**Supplemental Information**

**Part 1. Nanoparticle synthesis.**

The general scheme for the synthesis of zero-valent metal nanoparticles is shown in **Supplemental Figure 1**.

**
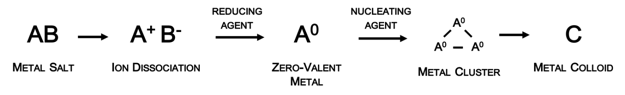
**

**Supplemental Figure 1.** General scheme for the synthesis of zero valent metal nanoparticles. The term “AB” represents a metal compound (*e.g.,* silver nitrate or copper (II) chloride). When suspended in the aqueous phase, the compound dissociates into ions. The addition of a reducing (*e.g.,* sodium borohydride) results in the metal cation losing its charge. The addition of a nucleating agent (*e.g.,* ethylene glycol) produces a “metal cluster”. Under controlled temperature and vigorous stirring for a specific duration, the cluster will grow in size and produce a metal colloid with stabilizing agent (*e.g.,* polyvinylpyrrolidone).

**Part 2. Nanoparticle microscopic characteristics.**

**Atomic force microscopy (AFM) was performed using Bruker Dimension Icon Atomic Force Microscope (**Santa Barbara, California, USA**) in tapping mode. The three-dimensional (3D) images are shown in Supplemental Figure 2.**


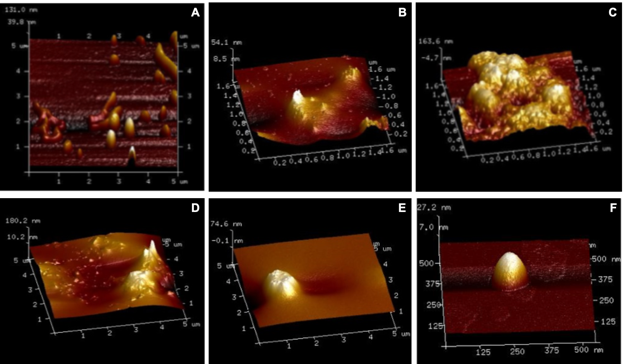


**Supplemental Figure 2.** Three dimensional images of the nanoparticles used in this study via AFM.

**Part 3. Nanoparticle spectroscopic characteristics.**

The ultraviolet-visible spectra of synthesized silver and copper nanoparticles are shown in **Supplemental Figure 3**. The silver particles show UV-Vis absorbance spectra with two absorbance peaks each, with Neg-AgNPs absorbance peaks at 250 nm and 450 nm, Neu-AgNPs absorbance peaks at 250 and 400 nm, and Pos-AgNPs absorbance peaks at 410 and 490 nm. The absorbance spectra of the synthesized copper particles display single absorbance peaks for Neg-CuNPs and Neu-CuNPs at 300 nm and Pos-CuNPs at 250 nm.

**Supplemental Figure 3.** The absorbance of light by nanoparticles is shown for wavelengths from the ultraviolet to the visible spectrum. The synthesized silver and copper nanoparticles are negatively, neutrally, or positively charged as a result of their stabilizing agent. The graphs show (A) silver nanoparticles coated with citrate (Neg-AgNPs), (B) silver nanoparticles coated with polyvinylpyrrolidone (Neu-AgNPs), (C) silver nanoparticles coated with cetyltrimethylammonium bromide (Pos-AgNPs), (D) copper nanoparticles coated with ascorbic acid (Neg-CuNPs), (E) copper nanoparticles coated with polyvinylpyrrolidone (Neu-CuNPs), and (F) copper nanoparticles coated with cetyltrimethylammonium bromide (Pos-CuNPs). Inserts are pictures of the nanoparticles after completion of the synthesis reaction.

The Fourier-transformed infrared (FTIR) spectra of AgNPs and CuNPs synthesized with charged and uncharged stabilizing agent are shown in **Supplemental Figure 4**. The spectra of Neg-AgNPs show O-H stretching bonds at 3648 cm^-1^, C=C stretch in the 1652 and 1558 cm^-1^ region as well as NO_2_ stretch in the 1558 cm^-1^ region. Neu-AgNPs showed N-H stretch and ≡C-H stretch in the 3249 cm^-1^ region and a C=C stretch in the 1635 cm^-1^ region. Pos-AgNPs spectra shows C=C stretch in the 1652 and 1558 cm^-1^ region, the C-H bend and NO_2_ stretch in the 1361 cm^-1^ region. Neg-CuNPs spectra shows an O-H stretching bond in the 3645 cm^-1^ region and a C=C stretch in the 1654 and 1557 cm^-1^ region. Neu-CuNPs shows peaks indictive of a shows C=C stretch in the 1652 and 1558 cm^-1^ region. Pos-CuNPs absorbance spectra shows N-H stretch and ≡C-H stretch in the 3248 cm^-1^ region and C=C stretch in the 1652 and 1558 cm^-1^ region.

**
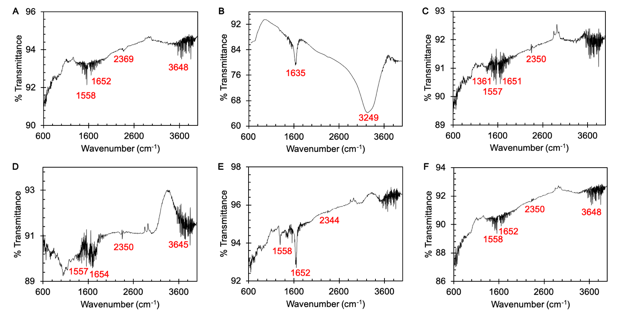
**

**Supplemental Figure 4.** The transmittance of light by nanoparticles is shown for wavenumbers in the infrared region of the spectrum. The synthesized silver and copper nanoparticles are negatively, neutrally, or positively surface charged as a result of their stabilizing agent. The graphs show (A) silver nanoparticles coated with citrate (Neg-AgNPs), (B) silver nanoparticles coated with polyvinylpyrrolidone (Neu-AgNPs), (C) silver nanoparticles coated with cetyltrimethylammonium bromide (Pos-AgNPs), (D) copper nanoparticles coated with ascorbic acid (Neg-CuNPs), (E) copper nanoparticles coated with polyvinylpyrrolidone (Neu-CuNPs), and (F) copper nanoparticles coated with cetyltrimethylammonium bromide (Pos-CuNPs).

**Part 4. Universal antibacterial properties.**

Bacterial tree of life with lineages highlighted to indicate divergence of the species used in this study, *E. coli* (Proteobacteria), *S. aureus* (Firmicutes), and *S. multivorum* (Bacteroidota), are depicted in **Supplemental Figure 5**.

*Escherichia* *coli*. *E.* *coli* is a gram-negative rod of the Enterobacteriaceae family and a facultative anaerobe with a negatively-charged cell surface. It is found in the gut [1]. Once it is expelled, the bacteria can live in water, sediment, and soil with or without oxygen. This is unlike other bacteria that are strictly anaerobic. *E. coli* morphology is small (*i.e.,* 2.5 μm in length and 0.8 μm in diameter). There are no nuclei, membrane-enclosed organelles, or any cytoskeletal elements. Cells have external organelles, pili (thin straight filaments), and flagella. Its integrity is held together with a thin, three-layered wall enclosing cytoplasm. Some strains cause urinary tract infections; others cause diarrheal diseases. On the other hand, common strains such as K-12 are helpful and protect the gut from excessive yeast and fungi growth. Biochemically, there are twenty-five (25) known gene products involved in promoting genetic exchange [2].

*Staphyloccocus* *aureus*. *S.* *aureus* is a gram-positive coci/bacuilli and is a member of the Staphylococceae family. It is also found in normal human flora, specifically in skin and mucous membranes. Morphologically, the bacterium is gram-positive, cocci-shaped, and arranged in clusters. It is resilient and can grow in water up to 10% salt content in either aerobic or anaerobic conditions. *S. aureus* does not cause infection on healthy skin; instead, it causes infection when it enters the bloodstream or internal tissues. It is associated with foodborne illnesses and infections in hospital settings. Biochemically, this bacterium has developed resistances to antibiotics (*e.g.,* MRSA (Methicillin-Resistant *Staphyloccus aureus*). There are some biochemical tests available to diagnosis infection: catalase positive is effective on all pathogenic *Staphylococcus* species; coagulase positive distinguishes *S. aureus* from other species; novobiocin sensitive (distinguishes *S. aureus* from *S. saprophyticus*; and mannitol fermentation positive distinguishes *S. aureus* from *S. epidermidis*.

*Sphingobacterium multivorum. S. multivorum* is member of the Sphingobacteriaceae family and is a non-fermentative gram-negative rod which produces catalase and oxidase. A characteristic feature of members of the Sphigobacteriaceae family is high concentrations of sphingolipids in the cell wall [3]. Sphingolipids are long fatty acid chains that play an important role in cell-cell interactions. Upon initial discovery of the *S. multivorum*, it was assumed that the bacteria were nonpathogenic. Later, it was determined that it can cause infection in immunocompromised humans [4]. *S. multivorum* can be isolated from the natural environment (*e.g.,* soil, plants, and water sources) or from the built environment (*e.g.,* hospitals and foods). Morphologically, its shape resembles straight rods that have no flagella but may exhibit sliding motility [5]. Biochemically, *S. multivorum* produces catalase and oxidase and is non-fermentative [6].

**
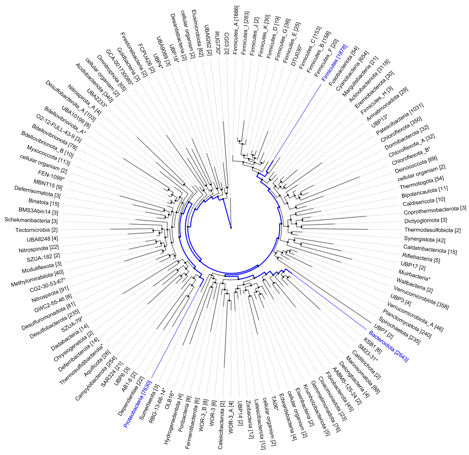
**

**Supplemental Figure 5.** Bacterial tree of life with lineages highlighted to indicate divergence of the species used in this study, *E. coli* (Proteobacteria), *S. aureus* (Firmicutes), and *S. multivorum* (Bacteroidota).

**REFERENCES**

1. Berg, H.C., *E. coli in Motion*. 2008: Springer Science & Business Media.

2. Kowalczykowski, S.C., et al., *Biochemistry of homologous recombination in Escherichia coli.* Microbiol. Mol. Biol. Rev., 1994. **58**(3): p. 401-465.

3. Prasad, S., et al., *Arcticibacter svalbardensis gen. nov., sp. nov., of the family Sphingobacteriaceae in the phylum Bacteroidetes, isolated from Arctic soil.* International journal of systematic and evolutionary microbiology, 2013. **63**(5): p. 1627-1632.

4. Mendes, M.D., et al., *Septic arthritis by Sphingobacterium multivorum in immunocompromised pediatric patient.* Revista Paulista de Pediatria, 2016. **34**(3): p. 379-383.

5. Yabuuchi, E., et al., *Sphingobacterium gen. nov., Sphingobacterium spiritivorum comb. nov., Sphingobacterium multivorum comb. nov., Sphingobacterium mizutae sp. nov., and Flavobacterium indologenes sp. nov.: glucose-nonfermenting gram-negative rods in CDC groups IIK-2 and IIb.* International Journal of Systematic and Evolutionary Microbiology, 1983. **33**(3): p. 580-598.

6. Abro, A.H., et al., *Sphingobacterium multivorum bacteremia and acute meningitis in an immunocompetent adult patient: a case report.* Iranian Red Crescent Medical Journal, 2016. **18**(9).
